# Effects of interoceptive accuracy on timing control in the synchronization tapping task

**DOI:** 10.3389/fnins.2022.907836

**Published:** 2023-01-06

**Authors:** Kenta Tomyta, Kentaro Katahira, Hideki Ohira

**Affiliations:** ^1^Department of Cognitive and Psychological Sciences, Graduate School of Informatics, Nagoya University, Nagoya, Japan; ^2^Japan Society for the Promotion of Science, Tokyo, Japan; ^3^National Institute of Advanced Industrial Science and Technology (AIST), Tsukuba, Japan

**Keywords:** interoception, rhythmic synchronization, interoceptive accuracy, tapping, electrocardiogram (ECG)

## Abstract

Humans often perform rhythmic synchronized movements. Professional musicians and dancers particularly perform such movement tasks well and have a higher interoceptive accuracy (IAcc) than non-musicians and non-dancers. We thus hypothesized that rhythmic synchronized movements might be enhanced by a higher IAcc. To investigate this hypothesis, this study conducted a heartbeat counting task and a rhythmic synchronization tapping task with normal (easier) and slow (harder) tempi metronomes. Inconsistent with our hypothesis, however, a higher IAcc was negatively correlated with timing control, but only in the slow tempo condition [*r* (30) = 0.46, *p* < 0.05]. This suggests that a higher IAcc did not enhance timing control in rhythmic synchronized movements but rather weakened it, resting heart rate variability was not correlated with timing control.

## Introduction

Sensorimotor synchronization, manifesting in activities such as dancing and playing music, is ubiquitous in our daily lives. Numerous studies have been conducted to investigate its underlying mechanism (see Repp, 2006; Repp and Su, 2013 for a review). Nevertheless, the psychological and neural bases of sensorimotor synchronization remain unclear in various respects. One such unresolved problem is the relationship between heartbeats and movement synchronized with external rhythm.

In a self-paced movement task, the rhythmic motor timing of the participant is affected by the rhythm of his or her own heart, which is known as cardiolocomotor synchronization (CLS; e.g., Kirby et al., 1991; Kirby, 1992; Takeuchi et al., 2015; De Bartolo et al., 2021). In a rhythmic synchronized movement task, participant needs to synchronize his or her movements to an external rhythm and not to his or her own heartbeat. However, considering CLS in self-paced movements, the timing of rhythmic synchronized movement would be affected to one’s own heartbeat. Although the CLS has already been described for decades, strangely, to our knowledge, no previous studies have investigated the direct relationship between heartbeat detection/perception ability and rhythmic synchronized movement. It is possible that only negative data have been obtained by unpublished previous studies focused on only movement entrainment to heartbeats.

Thus, this study aimed to investigate the relationship between heartbeat and rhythmic synchronized movement from the perspective of interoception, defined as the perception of internal bodily sensations from all organs (e.g., Craig, 2003). A recent study redefined interoception as the representation of the internal world, including the processes by which an organism senses, interprets, integrates, and regulates signals from within itself (Chen et al., 2021). One of the main topics in this research area is understanding how accurately we can perceive interoceptive signals (interoceptive accuracy; IAcc), such as the heartbeat (Schandry, 1981; Desmedt et al., 2020). Many studies have reported that a higher IAcc was positively correlated with other cognitive functions, such as decision-making, time perception and emotion recognition (Werner et al., 2009; Meissner and Wittmann, 2011; Terasawa et al., 2014).

Professional dancers and musicians have a higher IAcc than non-dancers and non-musicians (Schirmer-Mokwa et al., 2015; Christensen et al., 2018) and show good performance in rhythmic synchronized movement tasks (Repp, 2010; Miura et al., 2011). These findings indirectly imply that interoception enhances rhythmic synchronized movement. However, professional dancers and musicians do not only have a good rhythmic synchronization ability. For instance, music performance is composed of various elements, including perception of melody and rhythm, rhythmic motor control, and music knowledge (e.g., Thaut, 2013). In addition, Streeter et al. (1988) reported that participants with a low body fat percentage have a higher IAcc, and professional dancers could be considered to have a low body fat percentage. Thus, it is possible that the higher IAcc of dancers and musicians is correlated not with rhythmic movement ability but with other factors.

Here, this study directly investigated whether interoception enhances rhythmic synchronized movement. We used the synchronization tapping task with a metronome as a measure of the timing control of rhythmic synchronized movement and the heartbeat counting task as a measure of the IAcc (Schandry, 1981; Desmedt et al., 2020). As mentioned above, professional musicians and dancers have good rhythmic synchronization and IAcc (Schirmer-Mokwa et al., 2015; Christensen et al., 2018). Therefore, we hypothesized that the rhythmic synchronization tapping score would be positively correlated with that of the heartbeat counting task.

In addition, this study analyzed resting heart rate variability (HRV). Various indicators are used to analyze HRV. Fundamental measures are the standard deviation of R-R intervals (SDNN), the root mean square of successive heartbeat interval differences (rMSSDs) and the coefficient of variation of R-R intervals (CVrr). Frequency domain analysis is also conducted, in which R-R intervals were divided into very-low frequency (VLF; 0.0033–0.04 Hz), low frequency (LF, 0.04–0.15 Hz), and high frequency (HF, 0.15–0.4 Hz) (see section “Data analysis” for details). Lischke et al. (2021) showed that a higher HRV (rMSSD) was weakly correlated with a higher IAcc. In addition, previous studies in other research areas reported that a higher HRV (SDNN, frequency domain analyses) was correlated with other cognitive functions, such as memory and attention (Frewen et al., 2013; Williams et al., 2016). Based on these previous studies, HRV might be related to sensorimotor synchronization. Therefore, this study conducted an exploratory investigation of whether HRV is involved in sensorimotor synchronization. This study hypothesized that HRV would be positively correlated with timing control in the synchronization tapping task, consistent with the previous studies.

As mentioned above, the timing of self-paced rhythmic movement was affected by one’s own heart rate rhythm (Kirby et al., 1989; Kirby, 1992). Also, a movement timing in the non-rhythmic motor task was affected by one’s own heart rate rhythm. For example, on an electrocardiogram (ECG), the frequency of spontaneous motor timing increased around the T wave and decreased around the R wave (Palser et al., 2021). Thus, this study examined whether the frequencies of the metronome and of tapping were biased to a specific time window of the ECG (i.e., the timing of the heartbeat was entrained to that of the metronome, while the tapping of the tapping was entrained to that of the heartbeat).

## Materials and methods

### Participants

This study recruited 32 participants (13 men and 19 women, aged 18–37 years; mean = 21.88, SD = 3.47) who had not received dance training and were not professional musicians. The participants were Japanese undergraduate and graduate students. Although some participants had partaken of music training, Krause et al. (2010) detected no differences in rhythmic synchronization between amateur musician and non-musician participants. Therefore, the data of these 32 participants were pooled and analyzed as non-professional musicians or dancers. The main purpose of this study was to investigate the relationship between rhythmic synchronization movements and interoception. Therefore, correlation analysis was conducted for task scores between the heartbeat counting task and the synchronization tapping task. The preliminary experiment showed that the *r* coefficient was 0.5. Thus, we conducted sample size calculation based on the result. The calculation suggested a sample size of fewer than 29 was required (power of 0.8) at a significance level of 0.05 for the correlation analysis. This study was approved by the Ethics Committee of Nagoya University and conducted in accordance with the relevant guidelines (approval number NUPSY-210714-G-01).

### Procedures

At the beginning of the experiment, participants were required to fill out a questionnaire regarding personal data (e.g., age, sex, music, and dance experience). Then, a 10-min resting ECG was recorded, during which participants were required to fix their eyes on a fixation point approximately 60 cm in front of the face. Next, the heartbeat counting task was conducted, followed by the time estimation task. After conclusion of the two tasks, the participants were asked to report their usual resting heart rate (knowledge of the resting heart rate). Finally, they performed the synchronization tapping task.

### Tasks

#### Heartbeat counting task

Participants were asked to count their own heartbeats over three counting intervals (25, 35, and 45 s). The order of the three counting intervals was counterbalanced among participants. When “Start” was displayed on the screen with a cue sound, the participants were asked to start counting their heartbeats. When “End” was displayed on the screen with a cue sound, they were asked to end the count, after which they reported the number of heartbeats to the examiner. Importantly, they were prohibited from taking their pulse or using any other physical strategies to facilitate the counting of heartbeats during the task. Desmedt et al. (2020) showed that participants tended not to actually count their heartbeats but to estimate it using a time estimation and knowledge of their own resting heartbeat rate. To prevent the use of these strategies, participants were also instructed to report only the counts of the heartbeats they felt following the instructions in Desmedt et al. (2020). This protocol was based on the previous study (Desmedt et al., 2020).

#### Time estimation task

The procedure in the time estimation task was similar to that of the heartbeat counting task. The time estimation task also consisted of three counting intervals (28, 38, and 48 s), whose order was also counterbalanced among participants. When “Start” was displayed on the screen with a cue sound, the participants were instructed to start measuring the elapsed time. When “End” was displayed on the screen with a cue sound, the participants were instructed to stop, after which they reported their estimates. This protocol was based on the previous study (Desmedt et al., 2020).

#### Synchronization tapping task

The participants were instructed to synchronize the tapping of a wooden stick with a metronome (800 Hz pure tone) played from a set of speakers. The amplitude of the metronome signal was adjusted to level comfortable for the individual participant. This protocol was based on previous studies (e.g., Repp and Doggett, 2007; Tomyta and Seki, 2020). This task consisted of six conditions [metronome interonset interval (IOI) (BPM) = 462 ms (130), 600 ms (100), 857 ms (70), 1,000 ms (60), 1,200 ms (50), and 1,500 ms (40)], whose order was counterbalanced among the participants. In a session, the metronome signal was presented 300 times; that is, the participants synchronized their taps with the metronome 300 times in a row. During the task, an ECG was also recorded.

### Recordings

#### Electrocardiogram recordings

An ECG was recorded at 1,000 Hz with a BIOPAC MP 150 device (BIOPAC Systems Inc., Goleta, CA, USA) and AcqKnowledge software (BIOPAC Systems Inc., Goleta, CA, USA). R-peak detection was performed offline using Python (version 3.8).

#### Synchronization tapping task recordings

Participants were required to tap a plate (10.0 × 10.0 cm) attached to clips that were accessories from a CLIPHIT drum module (Korg, Tokyo, Japan), which served as a fine vibration sensor to detect the tap responses. The mean latency of the sensor between the tap and audio output was 0.33 ms (SD = 0.05 ms) (Tomyta and Seki, 2020). This enabled recording of the taps as sound pulses using an audio interface (Quad-Capture; Roland) and software (GarageBand, Apple), which were also used to record the metronome sounds. The recording system was based on the previous study (Tomyta and Seki, 2020). The onsets of the tapping and metronome sounds were detected offline using Python.

### Data analysis

#### Resting heart rate variability

The resting ECGs of 5 of the 32 participants were not properly recorded due to body motion, recording device problems and so on. Therefore, further analyses of the resting HRV were performed with the data of the remaining 27 participants. The R-R intervals were extracted from full 10-min resting ECG data with Python. SDNN, rMSSDs and CVrr were calculated as the index of the HRV. In addition, the R-R intervals were imported into Kubios HRV Standard software (Version 3.5.0; Niskanen et al., 2004) to obtain power spectrum density values. The VLF, LF, and HF power of the HRV was computed using the fast Fourier transform. The VLF-HRV reflects long-term regulation mechanisms, thermoregulation, and hormonal mechanisms (Laborde et al., 2017). The LF-HRV reflects a mix between sympathetic and parasympathetic tones (Laborde et al., 2017), and the HF-HRV reflects parasympathetic tone (Reyes del Paso et al., 2013). Additionally, the LF-HRV/HF-HRV ratio has been used as an index of sympathovagal balance (Forte et al., 2019).

However, the sympathovagal balance index calculated by the LF/HF HRV ratio is controversial. In particular, some studies have suggested that LF-HRV does not index sympathetic activity (e.g., [Bibr B14][Bibr B8]; Goldstein et al., 2011; Heathers, 2012; Billman, 2013; Reyes del Paso et al., 2013). For instance, Houle and Billman (1999) suggested that LF power probably results from an interaction of the sympathetic and parasympathetic nervous systems and does not accurately reflect changes in sympathetic activity. Additionally, Billman (2013) showed that the LF/HF ratio does not reflect sympathovagal balance. Therefore, we should interpret this result with caution. Nevertheless, it is worthwhile to examine whether HRV is related to rhythmic synchronized movement.

#### Heartbeat counting task

Following previous studies (e.g., Desmedt et al., 2020), the task score (IAcc) was calculated according to the standard formula: 1/3 Σ [1 − (| actual heartbeats − reported heartbeats|) / actual heartbeats]. A high IAcc score (maximum = 1) indicates that the participant performed well in the heartbeat counting task.

#### Synchronization tapping task

Generally, the IOIs of metronomes are usually less than 1,000 ms in synchronization tapping tasks (e.g., Lorås et al., 2012; Tomyta and Seki, 2020), whereas an IOI of more than 1,000 ms has often been defined as a “very slow tempo” (Repp and Doggett, 2007). Therefore, this study divided the 6 task conditions into normal (IOI = 462, 600, and 857 ms) and slow tempi conditions (IOI = 1,000, 1,200, 1,500 ms).

Ideally, 300 taps should have been obtained for each condition. However, the subjects occasionally failed to tap in time with the metronome. Therefore, a time window of 1/2 of the IOI of the metronome was set before and after metronome onset. The nearest tap onset to each metronome sound in the time window was regarded as a tapping response to the stimulus (e.g., Tomyta and Seki, 2020). When the tapping onset was outside the window, the tap was excluded from further analysis. Then, we calculated the synchronization error as the timing difference between the tap and metronome onsets [synchronization error (*n*) = tapping onset (*n*) − metronome onset (*n*)]. Then, the CV of the absolute value of the synchronization error was calculated.

#### Heartbeat entrainment in the synchronization tapping task

**FIGURE 1:**
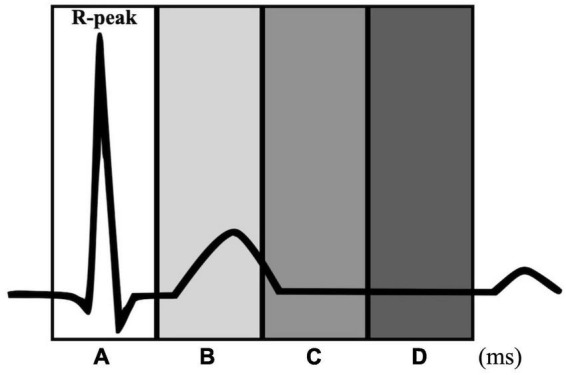
Time windows for the heartbeat entrainment analyses. Each window is defined relative to the R-peak: –100 to 100 ms (window A), 100 to 300 ms (window B), 300 to 500 ms (window C), and 500 to 700 ms (window D).

This study investigated whether the heartbeat was entrained to the metronome and the tapping entrained to the heartbeat using the methods described in Palser et al. (2021). We calculated the proportion of tapping and metronome events that occurred in four time windows relative to the R-peak: −100 to 100 ms (window A), 100 to 300 ms (window B), 300 to 500 ms (window C), and 500 to 700 ms (window D) (Figure 1).

### Statistical analysis

All analyses were conducted with R (version 4.1.0). For correlation analysis, Pearson correlation coefficients were computed between the reported heartbeat in the heartbeat counting task and the reported times in the time estimation task or knowledge of resting heart rate with Bonferroni correction. The correlation coefficients were also computed between the IAcc and the actual resting heartbeat.

Differences in the CV of the synchronization error and of the average tapping timing between the normal and slow tempi conditions were examined using the paired *t*-test. Differences in the heart rate in the resting state and during the synchronization tapping task were examined using one-way ANOVA.

Pearson correlation coefficients were also computed between the IAcc and the CV of the synchronization error in both the normal and slow tempi conditions with Bonferroni correction, between the HRV and CV of the synchronization error, and between the HRV and IAcc. Additional analysis was performed without dividing the CV of the synchronization error into normal and slow tempi conditions (see “Additional analysis between the IAcc and the CV of the synchronization error”).

To investigate whether there was any bias in the proportions of tapping and metronome events that occurred in the four time windows, we conducted one-way ANOVA (4 time windows) for each condition (normal tapping, normal metronome, slow tapping, and slow metronome). Moreover, we conducted a causal mediation analysis (CMA) to examine whether the bias in the proportions of tapping and metronome events meditated between the IAcc and the score of the synchronization tapping task.

## Results

### Validity of the heartbeat counting task in this study

The reported heartbeats in the heartbeat counting task were not correlated with the reported times in the time estimation task [*r* (30) = 0.17, *p* = 0.73] or with knowledge of the resting heart rate [*r* (30) = 0.25, *p* = 0.33]. Additionally, the IAcc was not correlated with the actual resting heart rate [*r* (26) = 0.22, *p* = 0.24]. These results suggest that the IAcc reported in this study reflects IAcc but not time estimation, knowledge of the resting heart rate or the actual resting heart rate.

### Synchronization tapping task

**FIGURE 2:**
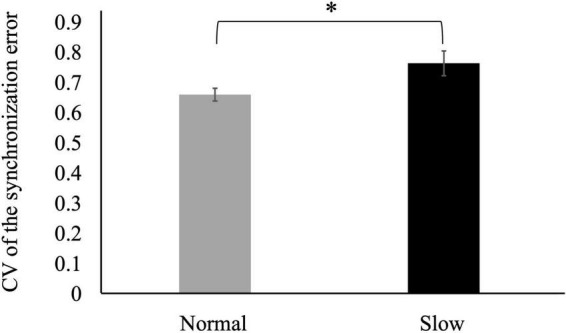
Coefficients of variation (CVs) of the synchronization error in the slow tempo condition were larger than those in the normal tempo condition (*p < 0.05). Error bars represent the standard error.

The CV of the synchronization error was larger in the slow tempo condition than in the normal tempo condition [*t* (31) = −2.23, *p* < 0.05] (Figure 2).

### R-R interval for heart rate in the resting state and the synchronization tapping task

**FIGURE 3:**
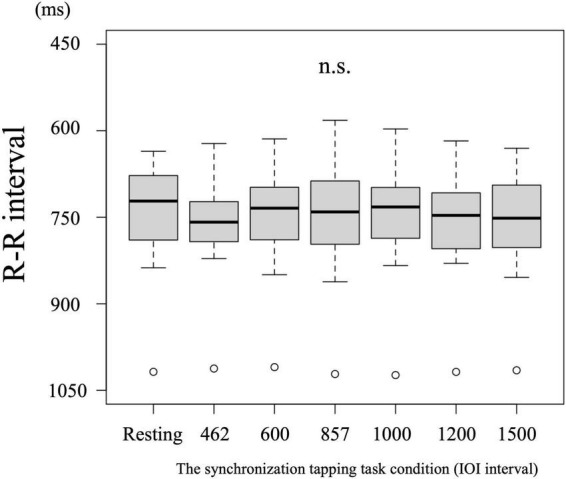
No differences were detected in the heart rate among the task conditions.

No differences were detected in the R-R interval among the resting state and the 462, 600, 857, 1,000, 1,200, or 1,500 ms IOI conditions [*F* (6,174) = 0.21, *p* = 0.97] (R-R interval: resting state = 0.736, 462 ms = 0.760; 600 ms = 0.746, 857 ms = 0.746, 1,000 ms = 0.741, 1,200 ms = 0.747, 1,500 ms = 0.757) (Figure 3).

### Correlation between the synchronization tapping task and the heartbeat counting task

**FIGURE 4:**
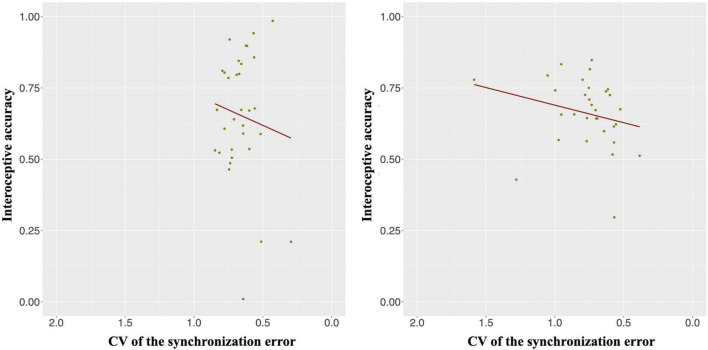
Interoceptive accuracy (IAcc) was positively correlated with the CV of the synchronization error in only the slow tempo (**right panel**; *r* = 0.46, *p* < 0.05) but not the normal tempo (**left panel**; *r* = 0.12, *p* = 1.00) condition. Inverting the values on the *x*-axis indicates that the farther the value is to the right, the higher the participant’s performance (lower variability).

The IAcc in the heartbeat counting task was positively correlated with the CV of the synchronization error in the synchronization tapping task in only the slow tempo but not the normal tempo condition [*r* (30) = 0.46, *p* < 0.05; *r* (30) = 0.12, *p* = 1.00] (Figure 4). In other words, the IAcc was negatively correlated with timing control in the synchronization tapping task (see Supplementary Table 1 for the descriptive statistics of IAcc and the CV of the synchronization error).

### Correlation between the synchronization tapping task and resting heart rate variability

The SDNN and rMSSD were not correlated with the CV of the synchronization error in either the normal [*r* (25) = 0.02, *p* = 1.00; *r* (25) = 0.08, *p* = 1.00] or the slow tempo condition [*r* (25) = −0.20, *p* = 0.63; *r* (25) = 0.03, *p* = 1.00]. The CVrr was not correlated with the CV of the synchronization error in either the normal or slow tempo condition [*r* (25) = 0.26, *p* = 1.00; *r* (25) = −0.38, *p* = 0.10]. The VLF, LF, HF, and LF/HF were also not correlated with the CV of the synchronization error in either condition [normal tempo condition: VLF, *r* (25) = 0.26, *p* = 0.38; VF, *r* (25) = −0.18, *p* = 0.72; HF, *r* (25) = 0.04, *p* = 1.00; VL/HF, *r* (25) = −0.16, *p* = 0.85; slow condition: VLF, *r* (25) = −0.07, *p* = 1.00; VF, *r* (25) = −0.39, *p* = 0.09; HF, *r* (25) = −0.05, *p* = 1.00; VL/HF, *r* (25) = −0.19, *p* = 0.69] (see Supplementary Table 2 for the descriptive statistics).

### Correlation between the heartbeat counting task and the resting heart rate variability

The SDNN and rMSSD were not correlated with the IAcc [*r* (25) = −0.03, *p* = 1.00; *r* (25) = −0.01, *p* = 1.00]. The CVrr was not correlated with the IAcc [*r* (25) = −0.32, *p* = 0.11]. The VLF, LF, HF, and LF/HF were not correlated with the IAcc [VLF, *r* (25) = −0.07, *p* = 0.73; VF, *r* (25) = −0.27, *p* = 0.18 HF, *r* (25) = 0.01, *p* = 0.96; VL/HF, *r* (25) = 0.01, *p* = 0.96].

### Heartbeat entrainment in the synchronization tapping task

The frequency of the metronome onsets was not biased in any of the ECG time windows in either the normal [*F* (3,99) = 0.63, *p* = 0.60] or slow tempo condition. [*F* (3,99) = 0.58, *p* = 0.63]. Additionally, the frequency of the tapping onsets was not biased in any of the ECG time windows in either the normal [*F* (3,99) = 1.64, *p* = 0.19] or slow tempo condition [*F* (3,99) = 0.09, *p* = 0.97].

### Causal mediation analysis

Causal mediation analysis of the frequency of the heartbeats entrained to the metronomes showed that the direct effect between the IAcc and the CV of the synchronization error was significant in the slow tempo condition (β = 0.65, *p* < 0.01, CI [0.25–1.06]) but not in the normal tempo condition (β = 0.23, *p* = 0.54, CI [−0.50 to 0.96]) (Supplementary Figure 1). The indirect effect from the CV to the frequency of the heartbeats entrained to the metronomes was not significant in either the normal or slow tempo condition (β = 0.03, *p* = 0.33, CI [−0.09 to 0.03]; β = −0.03, *p* = 0.12, CI [−0.07 to 0.03]). The indirect effect from the frequency of the heartbeats entrained to the metronomes to the IAcc was not significant in either the normal or slow tempo condition (β = −0.20, *p* = 0.93, CI [−4.59 to 4.18]; β = −2.17, *p* = 0.27, CI [−6.04 to 1.70]).

Causal mediation analysis of the frequency of tapping events entrained to the heartbeats showed that the direct effect between the IAcc and the CV of the synchronization error was significant in the slow tempo condition (β = 0.69, *p* < 0.001, CI [0.30–1.07]) but not in the normal tempo condition (β = 0.23, *p* = 0.53, CI [−0.48 to 0.94]). The indirect effect from the CV to the tapping events entrained to the heartbeats was not significant in either the normal or slow tempo condition (β = 0.01, *p* = 0.86, CI [−0.05 to 0.06]; β = 0.01, *p* = 0.57, CI [−0.03 to 0.06]). The indirect effect from the tapping events entrained to the heartbeats to the IAcc was not significant in either the normal or slow tempo condition (β = −1.98, *p* = 0.42, CI [−6.82 to 2.86]; β = 2.52, *p* = 0.10, CI [−0.51 to 5.56]). These results show that heartbeat entrainment did not mediate between the IAcc and the CV of the synchronization error.

## Discussion

This study investigated the relationship between rhythmic synchronized movement and interoception through administration of the synchronization tapping task and the heartbeat counting task. Contrary to our hypothesis, the main results showed a negative moderate correlation between the IAcc and the CV of the synchronization error in the slow but not the normal tempo condition. This result seems to contradict previous studies (Schirmer-Mokwa et al., 2015; Christensen et al., 2018), which reported that professional musicians and dancers have good IAcc. However, they are good not only at timing control but also in other abilities.

During the synchronization tapping task, the R-R interval was approximately 740 ms throughout all conditions. The interval belonged to the normal condition. The simplest reasoning is that heartbeat or interoception interacted with tapping performance in the normal tempo condition. However, our results showed a negative correlation between the IAcc and tapping performance only in the slow tempo condition. Therefore, we might consider that the difficulty of the task affected the results. Bååth and Madison (2012) showed that the variation in timing control increased as the metronome tempo slowed in a synchronization tapping task. Consistent with the findings of that study, the CV of the synchronization error was larger in the slow tempo condition than in the normal tempo condition, suggesting that the slow tempo condition was more difficult than the normal condition. Therefore, the difficulty of the task could explain the results of the correlation analysis between the CV of the synchronization error and the IAcc.

In the synchronization tapping task, the participants were asked to synchronize their movements to the sound of a metronome. Therefore, the movement timing should be affected by the presented metronome but not by their own heartbeat rhythm. Consistent with this speculation, this study showed that the frequency of the metronome sound and the tapping timing were not biased to any time window of the ECG (see next paragraph for details). However, considering that the timing of self-paced rhythmic movement is affected by one’s own heart rhythm (Kirby et al., 1991; Kirby, 1992), we assume that heartbeat rhythms affect motor control as a distractor in rhythmic synchronized movement, even if no obvious entrainment occurs. When the difficulty of the synchronization tapping task was low (normal tempo), it is possible that the participants blocked the interoceptive signal as a distractor for synchronized movement. On the other hand, when the difficulty of the synchronization tapping task was high (slow tempo), many cognitive resources were allocated for the task. As a result, the timing control of participants with higher IAcc would be affected by their own heartbeats in only the slow tempo condition. The reasoning for suppressing interoceptive signals is consistent with the competition of cues principle (e.g., Pennebaker, 1981), which proposes that internal signals are perceived more readily in the absence of distracting exteroceptive signals. In other words, when participants perceive exteroceptive signals, they need to suppress interoceptive signals. For instance, participants show less fatigue on the treadmill when they need to attend to exteroceptive information than when they need to attend to interoceptive attention (Pennebaker and Lightner, 1980). Based on the competition of cues principle, our reasoning of suppressing interoceptive signals is plausible. However, further studies are needed to examine this reasoning.

This study showed that the frequencies of the metronome and the tapping timing were not biased around the T wave, inconsistent with the results from Palser et al. (2021). In other words, the timing of the heartbeat was not entrained to that of the metronome, nor was the timing of the tapping entrained to that of the heartbeat. In addition, CMA revealed that the entrainment portions did not mediate between the IAcc and the CV of the synchronization error. Finally, no differences were detected in the heart rate among the conditions (resting state and 462 ms, 600 ms, 857 ms, 1000 ms, 1200 ms, and 1500 ms IOI conditions). These results suggest that heartbeat entrainment was not seen in the synchronization tapping task.

A previous study reported that the IAcc was negatively correlated with resting HRV (Lischke et al., 2021); however, no correlation was observed between these two indices in this study, possibly due to differences in the sample sizes between this study and that by Lischke et al. (2021). The sample size may be a reason why our results were inconsistent with those of a previous study in which the results showed a weak correlation between the IAcc and resting HRV in a cohort of more than 100 participants. In contrast, this study recruited 32 participants according to estimates from preliminary experiments and analysis of the required number of participants for correlation analysis for the scores between the heartbeat counting task and the synchronization tapping task. In addition, this study showed no correlation between the resting HRV and the timing control of the synchronization tapping task, but given the above sample size problem, this study cannot conclude that there is no relationship between the two. Thus, whether a higher HRV is associated with rhythmic synchronized movement remains an open question.

This study has several limitations. First, this study used the heartbeat counting task as an index of interoceptive accuracy. Participants generally tended to not actually count their heartbeats but to estimate them using time estimation and knowledge of their own resting heartbeat rate in this task, as mentioned in the Methods. In this study, we prevented participants from using these strategies with the improved instraction of Desmedt et al. (2020). However, this concern should be noted in interoception experiments. The heartbeat detection task (e.g., Kleckner et al., 2015) would also be useful. Ring and Brener (2018) suggested that the score in the heartbeat detection task was unrelated to that in the heartbeat counting task. Thus, further study is needed to investigate the relation between rhythmic synchronization movements and interoception with the heartbeat detection task. Moreover, it would be useful to use a questionnaire such as the multidimensional assessment of interoceptive awareness (MAIA; Mehling et al., 2012). Second, our experiment counterbalanced for only stimuli and/or conditions but not tasks (heartbeat counting task and synchronization tapping task). The structure of the experiment has been used in a previous study (e.g., Herman et al., 2021). For more detailed exploration, however, future studies should counterbalance the tasks.

## Conclusion

This study showed that participants with a higher IAcc had worse performance in the rhythmic synchronization tapping task than those with a lower IAcc. The results suggest that interoception becomes a distractor in the cognitive process when it is highly based on exteroception, such as the synchronization of rhythmic movement with external stimuli.

## Data availability statement

The original contributions presented in this study are included in the article/Supplementary material, further inquiries can be directed to the corresponding author.

## Ethics statement

The protocol was approved by the Ethical Committee of Nagoya University. The patients/participants provided their written informed consent to participate in this study.

## Author contributions

KT designed and carried out the study and wrote the manuscript. KT and KK analyzed the data. HO supervised the project. All authors contributed to the article and approved the submitted version.

## References

[B1] Bååth R. Madison G. (2012). “The subjective difficulty of tapping to a slow beat,” in *Proceedings of the 12th International Conference on Music Perception and Cognition*, (Thessaloniki).

[B2] Billman G. E. (2013). The LF/HF ratio does not accurately measure cardiac sympatho-vagal balance. *Front. Physiol.* 4:26. 10.3389/fphys.2013.00026 23431279 PMC3576706

[B3] Chen W. G. Schloesser D. Arensdorf A. M. Simmons J. M. Cui C. Valentino R. (2021). The emerging science of interoception: sensing, integrating, interpreting, and regulating signals within the self. *Trends Neurosci.* 44 3–16. 10.1016/j.tins.2020.10.007 33378655 PMC7780231

[B4] Christensen J. F. Gaigg S. B. Calvo-Merino B. (2018). I can feel my heartbeat: dancers have increased interoceptive accuracy. *Psychophysiology* 55:e13008. 10.1111/psyp.13008 28940488

[B5] Craig A. D. (2003). Interoception: the sense of the physiological condition of the body. *Curr. Opin. Neurobiol.* 13 500–505. 10.1016/S0959-4388(03)00090-4 12965300

[B6] De Bartolo D. De Giorgi C. Compagnucci L. Betti V. Antonucci G. Morone G. (2021). Effects of cognitive workload on heart and locomotor rhythms coupling. *Neurosci. Lett.* 762:136140. 10.1016/j.neulet.2021.136140 34324958

[B7] Desmedt O. Corneille O. Luminet O. Murphy J. Bird G. Maurage P. (2020). Contribution of time estimation and knowledge to heartbeat counting task performance under original and adapted instructions. *Biol. Psychol.* 154:107904. 10.1016/j.biopsycho.2020.107904 32464170

[B8] Eckberg D. L. (1997). Sympathovagal balance: a critical appraisal. *Circulation* 96 3224–3232. 10.1161/01.CIR.96.9.3224 9386196

[B9] Forte G. Favieri F. Casagrande M. (2019). Heart rate variability and cognitive function: a systematic review. *Front. Neurosci.* 13:710. 10.3389/fnins.2019.00710 31354419 PMC6637318

[B10] Frewen J. Finucane C. Savva G. M. Boyle G. Coen R. F. Kenny R. A. (2013). Cognitive function is associated with impaired heart rate variability in ageing adults: the Irish longitudinal study on ageing wave one results. *Clin. Autonomic Res.* 23 313–323. 10.1007/s10286-013-0214-x 24077752

[B11] Goldstein D. S. Bentho O. Park M. Y. Sharabi Y. (2011). Low-frequency power of heart rate variability is not a measure of cardiac sympathetic tone but may be a measure of modulation of cardiac autonomic outflows by baroreflexes. *Exp. Physiol.* 96 1255–1261. 10.1113/expphysiol.2010.056259 21890520 PMC3224799

[B12] Heathers J. A. (2012). Sympathovagal balance from heart rate variability: an obituary. *Exp. Physiol.* 97 556–556. 10.1113/expphysiol.2011.063867 22525665

[B13] Herman A. M. Esposito G. Tsakiris M. (2021). Body in the face of uncertainty: the role of autonomic arousal and interoception in decision-making under risk and ambiguity. *Psychophysiology* 58:e13840. 10.1111/psyp.13840 33977533

[B14] Hopf H. B. Skyschally A. Heusch G. Peters J. (1995). Low-frequency spectral power of heart rate variability is not a specific marker of cardiac sympathetic modulation. *Anesthesiology* 82 609–619. 10.1097/00000542-199503000-00002 7879929

[B15] Houle M. S. Billman G. E. (1999). Low-frequency component of the heart rate variability spectrum: a poor marker of sympathetic activity. *Am. J. Physiology-Heart Circulatory Physiol.* 267 H215–H223. 10.1152/ajpheart.1999.276.1.H215 9887035

[B16] Kirby R. L. (1992). The cardiac-locomotor coupling phenomenon: the contribution of Coleman. *Perceptual Motor Skills* 74 489–490. 10.2466/pms.1992.74.2.489 1594409

[B17] Kirby R. L. Gupta S. K. Carr S. E. MacLeod D. A. (1991). Cardiac-locomotor coupling while finger tapping: Part II. a cross-over control study. *Perceptual Motor Skills* 73 831–834. 10.2466/pms.1991.73.3.831 1792131

[B18] Kirby R. L. Nugent S. T. Marlow R. W. MacLeod D. A. Marble A. E. (1989). Coupling of cardiac and locomotor rhythms. *J. Appl. Physiol.* 66 323–329. 2917937 10.1152/jappl.1989.66.1.323

[B19] Kleckner I. R. Wormwood J. B. Simmons W. K. Barrett L. F. Quigley K. S. (2015). Methodological recommendations for a heartbeat detection-based measure of interoceptive sensitivity. *Psychophysiology* 52 1432–1440. 10.1111/psyp.12503 26265009 PMC4821012

[B20] Krause V. Pollok B. Schnitzler A. (2010). Perception in action: the impact of sensory information on sensorimotor synchronization in musicians and non-musicians. *Acta Psychol.* 133 28–37. 10.1016/j.actpsy.2009.08.003 19751937

[B21] Laborde S. Mosley E. Thayer J. F. (2017). Heart rate variability and cardiac vagal tone in psychophysiological research–recommendations for experiment planning, data analysis, and data reporting. *Front. Psychol.* 8:213. 10.3389/fpsyg.2017.00213 28265249 PMC5316555

[B22] Lischke A. Pahnke R. Mau-Moeller A. Weippert M. (2021). Heart rate variability modulates interoceptive accuracy. *Front. Neurosci.* 14:612445. 10.3389/fnins.2020.612445 33536870 PMC7849500

[B23] Lorås H. Sigmundsson H. Talcott J. B. Öhberg F. Stensdotter A. K. (2012). Timing continuous or discontinuous movements across effectors specified by different pacing modalities and intervals. *Exp. Brain Res.* 220 335–347. 10.1007/s00221-012-3142-4 22710620

[B24] Meissner K. Wittmann M. (2011). Body signals, cardiac awareness, and the perception of time. *Biol. Psychol.* 86 289–297. 10.1016/j.biopsycho.2011.01.001 21262314

[B25] Mehling W. E. Price C. Daubenmier J. J. Acree M. Bartmess E. Stewart A. (2012). The multidimensional assessment of interoceptive awareness (MAIA). *PLoS One* 7:e48230. 10.1371/journal.pone.0048230 PMC3486814 23133619

[B26] Miura A. Kudo K. Ohtsuki T. Kanehisa H. (2011). Coordination modes in sensorimotor synchronization of whole-body movement: a study of street dancers and non-dancers. *Hum. Movement Sci.* 30 1260–1271. 10.1016/j.humov.2010.08.006 21802159

[B27] Niskanen J. P. Tarvainen M. P. Ranta-Aho P. O. Karjalainen P. A. (2004). Software for advanced HRV analysis. *Comp. Methods Prog. Biomed.* 76 73–81. 10.1016/j.cmpb.2004.03.004 15313543

[B28] Palser E. R. Glass J. Fotopoulou A. Kilner J. M. (2021). Relationship between cardiac cycle and the timing of actions during action execution and observation. *Cognition* 217:104907. 10.1016/j.cognition.2021.104907 34563865 PMC8748943

[B29] Pennebaker J. W. (1981). Stimulus characteristics influencing estimation of heart rate. *Psychophysiology* 18, 540–548. 7280152 10.1111/j.1469-8986.1981.tb01824.x

[B30] Pennebaker J. W. Lightner J. M. (1980). Competition of internal and external information in an exercise setting. *J. Personal. Soc. Psychol.* 39:165. 10.1037/0022-3514.39.1.165 7411392

[B31] Repp B. H. (2006). Does an auditory distractor sequence affect self-paced tapping? *Acta Psychol.* 121 81–107. 10.1016/j.actpsy.2005.06.006 16098944

[B32] Repp B. H. (2010). Sensorimotor synchronization and perception of timing: effects of music training and task experience. *Hum. Movement Sci.* 29 200–213. 10.1016/j.humov.2009.08.002 20074825

[B33] Repp B. H. Doggett R. (2007). Tapping to a very slow beat: a comparison of musicians and nonmusicians. *Music Perception* 24 367–376. 10.1525/mp.2007.24.4.367

[B34] Repp B. H. Su Y. H. (2013). Sensorimotor synchronization: A review of recent research (2006–2012). *Psychon. Bull. Rev.* 20 403–452. 10.3758/s13423-012-0371-2 23397235

[B35] Reyes del Paso G. A. Langewitz W. Mulder L. J. Van Roon A. Duschek S. (2013). The utility of low frequency heart rate variability as an index of sympathetic cardiac tone: A review with emphasis on a reanalysis of previous studies. *Psychophysiology* 50 477–487. 10.1111/psyp.12027 23445494

[B36] Ring C. Brener J. (2018). Heartbeat counting is unrelated to heartbeat detection: a comparison of methods to quantify interoception. *Psychophysiology* 55:e13084. 10.1111/psyp.13084 29633292

[B37] Schandry R. (1981). Heart beat perception and emotional experience. *Psychophysiology* 18 483–488. 10.1111/j.1469-8986.1981.tb02486.x 7267933

[B38] Schirmer-Mokwa K. L. Fard P. R. Zamorano A. M. Finkel S. Birbaumer N. Kleber B. A. (2015). Evidence for enhanced interoceptive accuracy in professional musicians. *Front. Behav. Neurosci.* 9:349. 10.3389/fnbeh.2015.00349 26733836 PMC4681780

[B39] Streeter P. R. Rouse B. T. Butcher E. C. (1988). Immunohistologic and functional characterization of a vascular addressin involved in lymphocyte homing into peripheral lymph nodes. *J. Cell Biol.* 107 1853–1862. 10.1083/jcb.107.5.1853 2460470 PMC2115336

[B40] Takeuchi S. Nishida Y. Mizushima T. (2015). Evidence of an association between cardiac-locomotor synchronization and lower leg muscle blood perfusion during walking. *J. Phys. Therapy Sci.* 27 1819–1822. 10.1589/jpts.27.1819 26180328 PMC4499991

[B41] Terasawa Y. Moriguchi Y. Tochizawa S. Umeda S. (2014). Interoceptive sensitivity predicts sensitivity to the emotions of others. *Cogn. Emot.* 28 1435–1448. 10.1080/02699931.2014.888988 24559130

[B42] Thaut M. (2013). *Rhythm, Music, and the Brain: Scientific Foundations and Clinical Applications.* London: Routledge. 10.4324/9780203958827

[B43] Tomyta K. Seki Y. (2020). Effects of motor style on timing control and EEG waveforms in self-paced and synchronization tapping tasks. *Neurosci. Lett.* 739:135410. 10.1016/j.neulet.2020.135410 33091439

[B44] Werner N. S. Jung K. Duschek S. Schandry R. (2009). Enhanced cardiac perception is associated with benefits in decision-making. *Psychophysiology* 46 1123–1129. 10.1111/j.1469-8986.2009.00855.x 19558399

[B45] Williams D. P. Thayer J. F. Koenig J. (2016). Resting cardiac vagal tone predicts intraindividual reaction time variability during an attention task in a sample of young and healthy adults. *Psychophysiology* 53 1843–1851. 10.1111/psyp.12739 27658566

