# Supplementary material for: Effects of interoceptive accuracy on timing control in the synchronization tapping task
Source: Front Neurosci. 2023 Jan 6;16:907836. doi: 10.3389/fnins.2022.907836 (PMC9853000; doi:10.3389/fnins.2022.907836)
Supplement: Supplementary file 1 [file Data_Sheet_1.docx]

**Additional analysis between the IAcc and the CV of the synchronization error**

The IAcc in the heartbeat counting task was positively correlated with the CV of the synchronization error in the synchronization tapping task at only 1500 ms of the IOI condition (r (30) = 0.52, *p* <.05) but not at 462 ms, 600 ms, 857 ms, 1000 ms, or 1200 ms (r (30) = 0.15, *p* = 1.00, r (30) = 0.10, *p* = 1.00, r (30) = 0.24, *p* = 1.00, (r (30) = 0.35, *p* =.30).

Table. S1. Descriptive statistics of IAcc and CV of the synchronization tapping task.


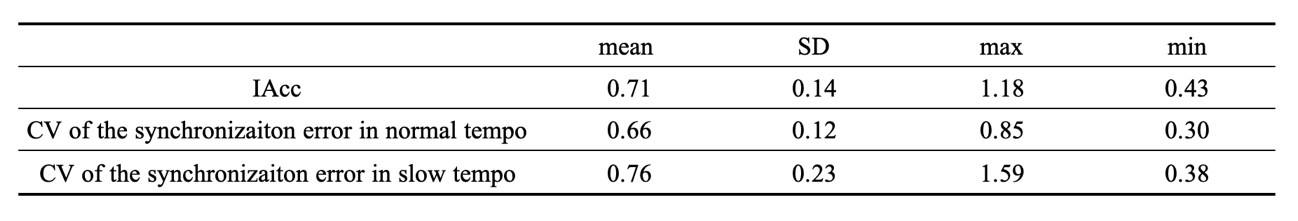


Table. S2. Frequency domain analyses for heart rate variability


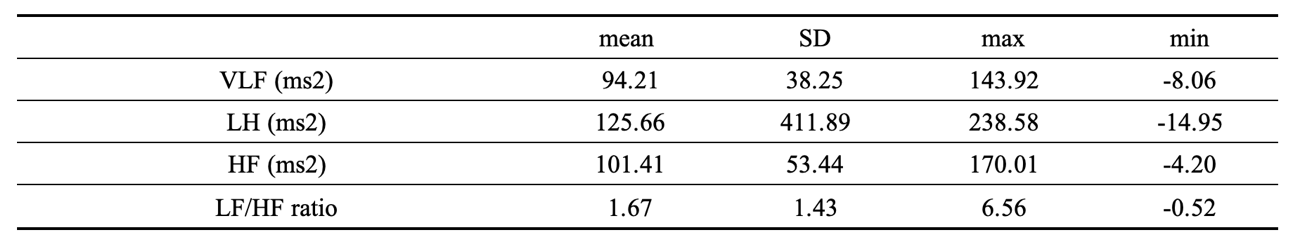


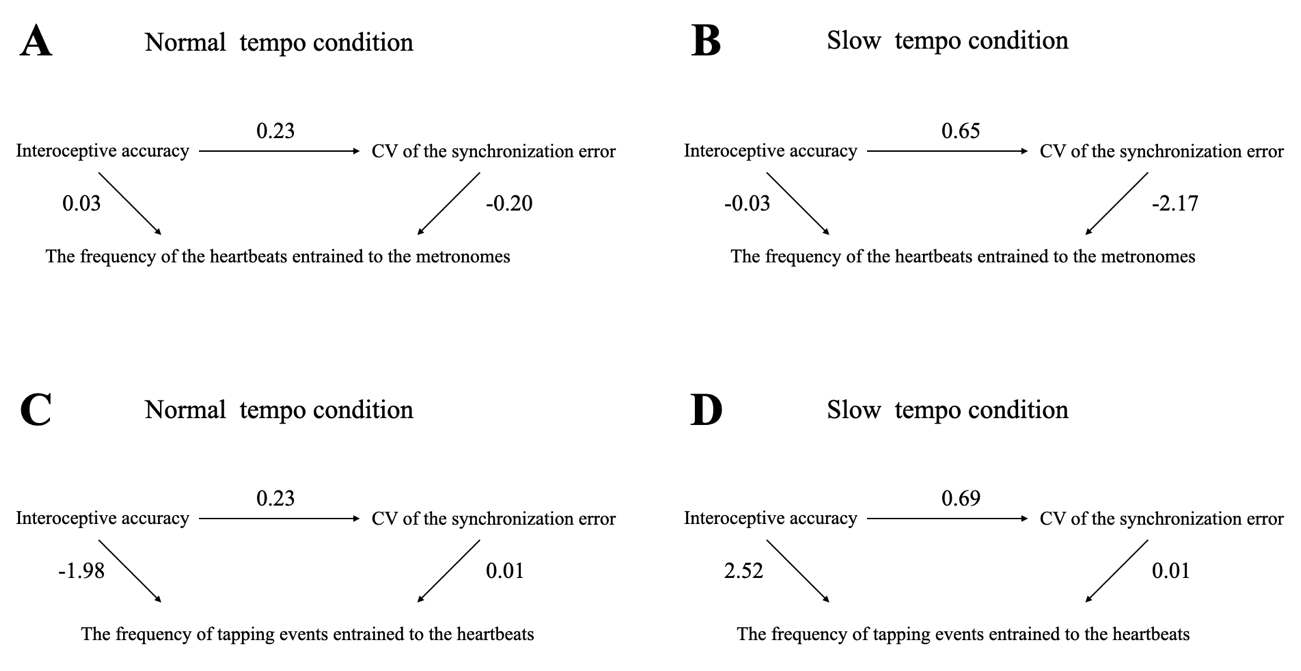


Fig S1. Graphs of the mediation model
